# HIV-1 gp120-mediated increases in IL-8 production in astrocytes are mediated through the NF-κB pathway and can be silenced by gp120-specific siRNA

**DOI:** 10.1186/1742-2094-7-96

**Published:** 2010-12-29

**Authors:** Ankit Shah, Anil Kumar

**Affiliations:** 1 Division of Pharmacology and Toxicology, UMKC-School of Pharmacy, Kansas City, MO 64108, USA

## Abstract

**Background:**

The exact mechanism underlying HIV-associated neurocognitive disorders still remains largely unresolved. However, viral genes (for example *gp120 *and *tat*) and their effect on cytokine/chemokine expressions have been linked with neuroinflammation. Conversely, interlekin-8 (IL-8) is a known proinflammatory chemokine and is known to be over-expressed in human brain microvascular endothelial cells in response to gp120. In this study, we sought to address whether HIV-1gp120 could affect IL-8 expression in astrocytes and whether the NF-κB pathway is involved in this phenomenon.

**Methods:**

SVGA astrocytes were transfected with a plasmid expressing HIV-1 pSyn gp120 JR-FL using Lipofectamine2000. The cells were harvested at different time points after transfection, and total cellular RNA was used for quantification of IL-8 using a real time PCR. IL-8 protein expression was also determined in supernatants collected at different time points after transfection. Involvement of the NF-κB pathway was addressed using both pharmacological inhibitors and an siRNA approach. In order to explore gene specificity, gp120-specific siRNAs were designed and IL-8 expression was monitored at both mRNA and protein levels.

**Results:**

Gp120 increased IL-8 expression both at mRNA and protein levels by 7.1 ± 1.04 and 2.41 ± 0.35 fold at 6 and 48 hours post-transfection, respectively. This increase was time-dependent and was abrogated by use of gp120-specific siRNA. We have also shown that the NF-κB pathway is involved in gp120-mediated IL-8 overexpression as IKK-2 and IKKβ inhibitors inhibited IL-8 expression by 63.5% and 57.5%, respectively at the mRNA level, and by 67.3% and 58.6% at the protein level. These results were also confirmed with use of NF-κB-specific siRNA.

**Conclusion:**

These results indicate that gp120 can modulate expression of a pro-inflammatory chemokine (IL-8) in astrocytes in a time-dependent manner with significant up-regulation at different times. This phenomenon is specific and is mediated by the NF-κB pathway.

## Background

Human immunodeficiency virus (HIV-1) can cause infection in the central nervous system (CNS) of an infected individual and is responsible for HIV-associated neurocognitive disorder (HAND). Gp120, a surface glycoprotein, not only plays an important role in attachment and viral entry [1-3] into host cells but is also known to cause neurotoxicity through a variety of mechanisms. These include oxidative stress [4], white matter gliosis, loss of the structural integrity of blood brain barrier (BBB) [5] and neuronal cell loss [6]. These types of neurological damage, especially gliosis and inflammation in the brain, have been found to correlate with increased production of proinflammatory cytokines/chemokines [7-10].

The astrocyte is a major CNS cell type and is known to exhibit limited productive replication of the virus [11]. Astrogliosis has also been very commonly reported in brain of infected patients [12]. The viral protein gp120 has been shown to be directly correlated with increased production of TNF-1α, IL-1β and IL-6; and is inversely correlated with expression of P-glycoprotein in rat astrocytes [13,14]. Furthermore gp120 has also been shown to increase IL-6 production in mixed human brain cell culture [15].

Interleukin (IL)-8 is an important chemokine, which responds in combination with other inflammatory mediators [16,17]. It has been reported to be increased during brain injury and neuroinflammation [18]. HIV-1 tat has been shown to induce IL-8 in human brain-derived endothelial cells and astrocytes [19,20]. Furthermore, IL-8 has also been reported to be involved in a STAT1-dependent mechanism for gp120-mediated increased IL-8 production in human brain microvascular endothelial cells [21]. Thus, together all of these studies suggest a potential role for IL-8 in HIV-associated neuroinflammation. However, there is no direct evidence as to whether gp120 would cause IL-8 expression in astrocytes.

In this study, we sought to address the question as to whether gp120 would affect IL-8 expression in a human astrocytic cell line, SVGA. We also sought to address whether the NFkB pathway is involved in this process, and this was accomplished using NFkB inhibitors and siRNA.

## Methods

### Cells and reagents

SVGA is a clone of a human fetal astrocyte cell line (SVG) [22] and was maintained in Dulbecco's Modified Eagle Medium (DMEM) supplemented with 10% fetal bovine serum (FBS) and 50 μM gentamicin at 37°C in 5% CO_2 _environment. Lipofectamine™ 2000, and NF-kB inhibitors (IKK-2; SC514 and IKK-β; BAY117082) were obtained from Invitrogen Inc. (Carlsbad, CA) and Calbiochem (EMD Biosciences Inc., La Jolla, CA), respectively. The HIVgp120 plasmid (Cat number 4598; pSyn gp120 JR-FL) was originally developed by Drs. Park and Seed [23], and was obtained from NIAID AIDS Reagent Center. Gp120-specific small interfering RNA (siRNA) was designed using SDSC Biology Workbench software, and various sequences of the siRNA targeted against gp120 were commercially synthesized by Ambion Inc. (Applied Biosystems, Foster city, CA). Pre-designed siRNA for NF-kB (P/N AM51331; id 5213) and Rel-A (P/N 4390824; id s11914) were also purchased from Ambion Inc. (Applied Biosystems, Foster city, CA).

### Transfection

SVGA cells were transfected with Lipofectamine™ 2000 as recommend by the manufacturer. Briefly, 1 × 10^6 ^cells were transiently transfected with 2 μg gp120 and 4 μl of lipofectamine in 1 ml serum-free medium for a period of 5 hours. The cells were harvested and total RNA was extracted using RNeasy kit from Qiagen (Valencia, CA). IL-8 expression was measured at time points of 6, 12, 24, 48 and 72 hours after the transfection. For NF-kB inhibition experiments, the cells were treated with 10 μM antagonist for 24 hours prior to the transfection. siRNA transfection was also performed using Lipofectamine™ 2000 and incubation for 48 hrs prior to gp120 transfection. Briefly, 50 nmoles of siRNA was transfected into each well containing 1 × 10^6 ^astrocytes in serum-free media. The transfection media was replaced after 24 hours with fresh DMEM supplemented with 10% FBS and the cells were incubated for 24 hours. Later, the cells were transiently transfected with gp120 as described above and the IL-8 levels were monitored 6 hours post-transfection. Inhibition of IL-8 expression was calculated as a percentage of expression in controls that were either mock-transfected or transfected with scrambled siRNA.

### Real time RT-PCR and IL-8 protein assay

Real time reverse transcriptase polymerase chain reaction (RT-PCR) was used to measure IL-8 mRNA using forward primer (5' CTC TTG GCA GCC TTC CTG ATT 3'), reverse primer (5' TAT GCA CTG ACA TCT AAG TTC TTT AGC A 3'), and probe (5' FAM-CTT GGC AAA ACT GCA CCT TCA CAC AGA-3' BHQ) in a Bio-Rad iCycler. Total RNA was extracted using RNeasy kit (Qiagen, Valencia, CA) as described in the manufacturer's protocol. Reverse transcription and amplification was done using 150 ng RNA as template in 25 μl reaction volume containing reverse transcriptase, primers, probe, DNA polymerase and other reagents. The reaction conditions included reverse transcription at 50°C for 30 min, initial PCR activation at 95°C for 15 min and 50 cycles of denaturation at 95°C for 30 sec and annealing at 61°C for 30 sec. Separate hypoxanthine-guanine phosphoribosyl-transferase (HPRT - house-keeping gene) amplification was used determine fold difference in expression. The data was analyzed using the equation 2^-ΔΔCT ^method as described previously [24].

Cell culture supernatant was collected after the transfection at different times and IL-8 protein concentrations were analyzed using a Bio-Plex System (Life Science Research, Hercules, CA). The protein expression was measured by comparing the values with the 5PL-standard curve using Bio-Plex Manager 5.0 software.

### Statistical analysis

Data are expressed in mean ± SE for 3 experiments with each experiment done in triplicates. The statistical significance was calculated using student's t test, and a p value ≤ 0.05 was considered significant.

## Results

### HIV-1 gp120 induces the expression of IL-8 in time dependent manner

**Figure 1:**
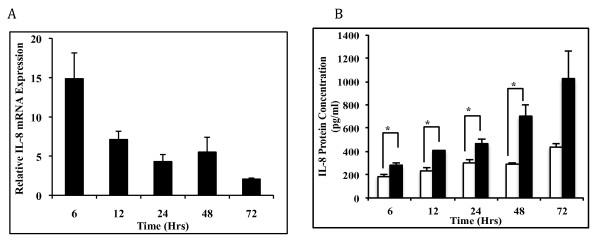
**Gp120-mediated increased expression of IL-8 in SVGA astrocyte cells**. 1 × 10^6 ^SVGA astrocytes were transfected with 1 μg gp120 DNA for 5 hours, using a lipofection method. Total RNA was extracted from cells at 6, 12, 24, 48 and 72 hours after transfection, and culture supernatant was collected at the same times. IL-8 mRNA was determined using real time PCR whereas protein was measured using a Bio-plex method. Expressions of IL-8 at the mRNA (A) level were compared between gp120-transfected cells and those with empty plasmids, and are presented as fold change. (B) IL-8 protein levels are shown as pg/ml culture supernatant and were compared between gp120-transfected and control cells transfected with empty plasmid. Each bar represents mean ± SE for 3 independent experiments, with each experiment done in triplicate. Student's t test was used for statistical analysis and statistical significance is denoted as * (p value ≤ 0.05).

Interleukin- (IL-) 8 is a major pro-inflammatory chemokine, which has been associated with neuroinflammatory mechanisms as well. However, what role gp120 plays in the expression levels of IL-8 is still unclear. Firstly, we wanted to ascertain whether astrocytes increase IL-8 expression upon gp120 transfection. SVGA cells were transfected with a gp120-expressing plasmid using Lipofectamine2000 reagent. Our transfection efficiency has been between 55 and 70%, which was confirmed with GFP transfection followed by analysis using FACScanto flowcytometer (data not shown). IL-8 mRNA and protein expressions were monitored at 6, 12, 24, 48 and 72 hrs post-transfection (Figure 1). Peak IL-8 mRNA level was observed at 6 hrs (14.9 ± 3.27 fold) and this diminished in a time-dependent fashion from this peak level. IL-8 mRNA was found to be 7.1 ± 1.04, 4.3 ± 0.89, 5.5 ± 1.9 and 2.1 ± 0.14 folds after 12, 24, 48 and 72 hour post-transfection, respectively (Figure 1A).

We further measured the protein expression in supernatants collected at different times after transfection with gp120. There was significant increase in IL-8 protein in gp120-transfected astrocytes at 6 hours as compared to mock-transfected control (279.7 ± 22.6 vs 183.9 ± 22.6 pg/ml). The concentration gradually increased in both control and gp120-transfected cells. However, gp120-transfected cells showed a much higher and significant increase in IL-8 expression with 407.8 ± 3.7, 464.2 ± 39.5, 700.8 ± 101.5 and 1022.7 ± 232.9 pg/ml at 12, 24, 48 and 72 hours, respectively (Figure 1B). During these times, IL-8 expression was 1.52 to 2.41 fold higher in gp120-transfected cells as compared to those in control wells and the differences between gp120 and control were statistically significant (p ≤ 0.05) at 6, 12, 24 and 48 hours post-transfection.

### Gp120-specific small interfering RNA (siRNA) significantly abrogated IL-8 expression

**Figure 2:**
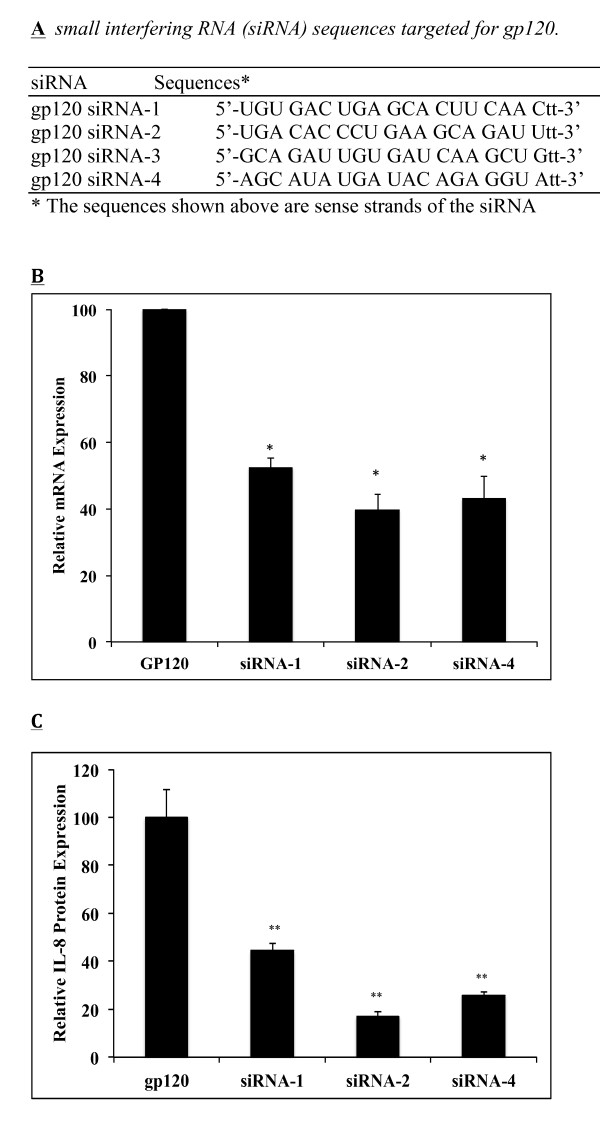
**Inhibition of gp120-induced IL-8 expression by gp120-specific siRNA**. Four different siRNAs for gp120 were designed using the Ambion siRNA designing tool, and were commercially synthesized by Ambion Inc (A). 1 × 10^6 ^astrocytes were transfected with 50 nM of siRNA for 48 hours followed by gp120 transfection. mRNA (B) and protein (C) expressions of IL-8 were measured at 6 and 48 hours post gp120 transfection, respectively. mRNA expression is represented as fold change between gp120 transfected cells and cells transfected with empty plasmid. The protein expressions are presented as absolute concentrations (pg/ml). Each bar represents mean ± SE for 3 independent experiments with each experiment done in triplicate. Student's t test was used for statistical analysis and statistical significance is denoted as * (p value ≤ 0.05).

In next series of experiments, we sought to determine whether this IL-8 over-expression was specific to gp120. For the purpose, we designed 4 siRNA sequences for gp120 as shown in Figure 2A. The siRNAs were transfected into SVGA using Lipofectamine2000 for 48 hours prior to gp120 transfection. mRNA and protein levels of IL-8 were estimated as mentioned before. All of them except siRNA-3 efficiently silenced gp120-mediated IL-8 expression to different degrees, both at mRNA and protein levels. SiRNA1, 2 and 4 inhibited by 47, 60 and 57% IL-8 expression at the mRNA level (6 hours post-transfection) and by 56, 83 and 74% inhibition at the protein level (48 hours post-transfection). As shown in Figure 2B and 2C, siRNA-2 was the most potent at inhibiting gp120-mediated IL-8 expression, both at the mRNA and protein levels. However, inhibitions at protein levels were higher than those at mRNA levels.

### gp120 mediated increase in IL-8 expression was abrogated with NF-κB specific antagonists and siRNA

**Figure 3:**
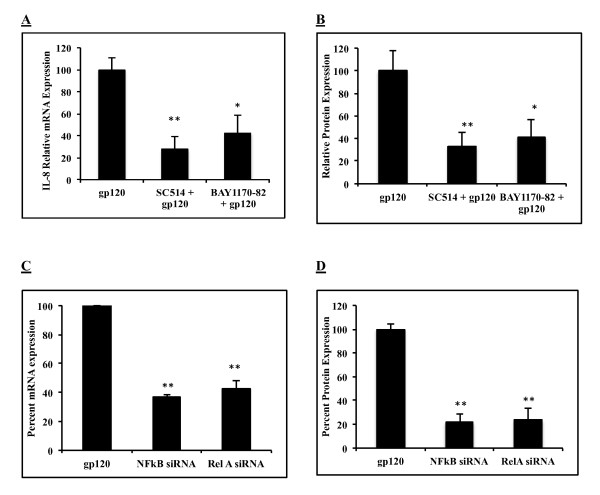
**Inhibition of gp120-mediated IL-8 expression by pharmacological inhibitors and siRNAs specific for the NF-κB pathway**. SVGA astrocytes were treated with 10 μM of SC514 (IKK-2 Inhibitor) and BAY11-7082 (IKKβ) for 24 hours followed by gp120 transfection in the presence of inhibitor. The appropriate inhibitor concentration was maintained throughout experiment. The relative IL-8 mRNA expressions (A) and protein expressions (B) are expressed as a percentage of levels in inhibitor-treated gp120-transfected cells relative to untreated cells. For siRNA experiments, SVGA astrocytes were transfected with NF-κB pathway-specific siRNAs for 48 hours followed by gp120 transfection. mRNA and protein levels were determined at 6 and 48 hours post-transfection, respectively. Inhibition of IL-8 mRNA (C) and protein (D) are presented as a percentage of IL-8 expression in gp120-transfected cells. Each bar represents mean ± SE for 3 independent experiments with each experiment done in triplicates. Student's t test was used for statistical analysis and statistical significance is denoted as * (p value ≤ 0.05).

Our next question was to determine whether the NF-κB pathway was involved in the gp120-mediated increase in IL-8 levels. We tested this by using two chemical inhibitors and two unique commercially available siRNAs. SVGA astrocytes were treated with 10 μM of IKK-2 (SC514) and IKKβ (BAY11-7082) inhibitors for 24 hours prior to transfection with gp120, and maintained with inhibitors throughout the experiment. IL-8 mRNA and protein expressions were monitored 6 and 48 hours after transfection respectively. Both SC514 and BAY11-7082 successfully inhibited gp120-mediated expression of IL-8 mRNA by 27.5 ± 11.5% and 42.5 ± 15.9% respectively, (Figure 3A). Similarly, IL-8 protein level was also reduced by 67.3 ± 12.6% and 58.6 ± 15.4%, respectively by SC514 and BAY11-7082 (Figure 3B).

We also tested 2 commercially available siRNAs against NF-κB1 and Rel-A to determine their effects on gp120-mediated IL-8 regulation. Specific siRNA for NFkB-1 and Rel A were transfected into astrocytes 48 hours prior to gp120 transfection, and mRNA/protein expressions were measured as described above. Both NF-κB- and Rel-A-specific siRNA blocked >55 and >75% of mRNA and protein expression, respectively (Figure 3C &3D). These experiments unambiguously confirm the results from chemical inhibition experiments that gp120-mediated increases in IL-8 expression are dependent on the NF-κB pathway.

## Discussion

HIV-1 glycoprotein 120 (gp120) plays an essential role in viral attachment and entry into host cells. In this study, we showed that gp120 induces IL-8 expression at both RNA and protein level in a time-dependent manner in astrocytes. Several earlier reports from different laboratories have shown the neurotoxic potential of gp120 during HIV-1 infection [25,26]. This phenomenon has also been shown to be mediated via oxidative stress [13,27], and has also been shown to correlate with increased production of TNF-α, IL-1β and IL-6 [9,28,29]. Recently, an elegant report by Li and co-workers showed indirect evidence for a role for gp120 in IL-8 over-expression, wherein they showed reduced IL-8 production in astrocytes when these were infected with gp120-deleted virion compared to those cells that were infected with wild-type [12].

In this study we have for the first time shown that gp120 is involved in IL-8 up-regulation in time-dependent manner, and that this effect is specific. This was confirmed by using gp120-specific siRNA. The siRNA approach has evolved into an important tool for gene-silencing studies in mammalian systems during recent years, and a variety of siRNAs have been used to knockdown various genes like gag, tat, pol and integrase, and to inhibit HIV replication [30-34]. However, no study has reported inhibition of IL-8 or any other cytokine/chemokine using the siRNA approach. In order to assess whether IL-8 induction is indeed a result of gp120 introduction into cells, we used various siRNAs in order to knockdown gp120 expression. In view of significant inhibition observed both at RNA and protein levels, we conclude that IL-8 induction observed in gp120-transfected cells is gene specific.

In this study, we have shown that gp120-mediated increases in IL-8 expression could be inhibited by specific NF-κB pathway inhibitors, implying role for this pathway in IL-8 production. Earlier studies have already shown a role for NF-κB activation in IL-8 production in asthmatic patients [35]. Using various mutational and deletion analysis it has been proved that certain promoter elements in NF-κB complex play a potential role in inducing the IL-8 promoter, which emphasizes the important role of NF-κB in IL-8 transcription [36,37]. SC514 is a novel inhibitor of NF-κB, which targets IKK-2 [38], whereas BAY11-7082 blocks NF-κB activation by inhibiting TNF-α-induced phosphorylation of IKKβ [39]. IkKβ acts as a negative regulator of the NF-κB pathway and prevents NF-κB activation. Phosphorylation of IkKβ leads to activation of NF-κB, and activated NF-κB later incorporates into the nucleus and leads to a downstream cascade. In our study, we observed that inhibition of NF-κB activation by targeting IkKβ leads to partial abrogation of gp120-mediated IL-8 expression. Involvement of the NF-κB pathway was also confirmed by silencing the NF-κB gene, which resulted in partial-to-near-complete restoration of gp120-mediated IL-8 expression to basal level in astrocytes.

In summary, we have shown that gp120 induces up-regulation of IL-8 in astrocytes, and that the NF-kB pathway appears to be predominantly responsible for this since direct interference with this pathway disrupts gp120-dependent induction of IL-8. We have also shown that gp120-specific siRNA abrogates this effect, suggesting that IL-8 over-expression is gp120 specific. These data suggest that IL-8 may be a potential target for intervention to reduce or ameliorate neuroinflammation, and could also become an important adjunct therapeutic strategy for future consideration.

## Competing interests

The authors declare that they have no competing interests.

## Authors' contributions

AS performed all the experiments and prepared first draft of the manuscript. AK designed the project, supervised AS throughout experimental phase and finalized the manuscript. All authors have read and approved the final manuscript.
